# Predicting the benefits of type-2 targeted anti-inflammatory treatment with the prototype Oxford Asthma Attack Risk Scale (ORACLE)

**DOI:** 10.1183/23120541.00570-2021

**Published:** 2021-02-07

**Authors:** Simon Couillard, William Il Hoon Do, Richard Beasley, Timothy S.C. Hinks, Ian D. Pavord

**Affiliations:** 1 Respiratory Medicine Unit and Oxford Respiratory NIHR BRC, Nuffield Dept of Medicine, University of Oxford, Oxford, UK; 2 Faculté de Médecine et des Sciences de la Santé, Université de Sherbrooke, Sherbrooke, QC, Canada; 3 Medical Research Institute of New Zealand, Wellington, New Zealand

## Abstract

Reduction of the risk of severe asthma attacks is a major goal of current guidelines [1]. The observation that blood eosinophils and exhaled nitric oxide fraction (*F*_ENO_) identify the higher risk type-2 inflammatory phenotype in asthma is potentially relevant to this goal [2].

## Abstract 2

**The prototype ORACLE scale based on two simple measures of type 2 airway inflammation (blood eosinophils and *F*_ENO_) quantifies the excess risk conferred by raised biomarkers that is removed by type-2 anti-inflammatory treatment in trial populations** https://bit.ly/3F1gnUl

*To the Editor:*

Reduction of the risk of severe asthma attacks is a major goal of current guidelines [1]. The observation that blood eosinophils and exhaled nitric oxide fraction (*F*_ENO_) identify the higher risk type-2 inflammatory phenotype in asthma is potentially relevant to this goal [2].

Blood eosinophils and *F*_ENO_ provide complementary mechanistic information on different immune compartments and inflammatory mediators involved in the pathogenesis of asthma: whereas blood eosinophils reflect the systemic pool of effector cells and circulating interleukin-5, *F*_ENO_ identifies type-2 inflammation in the airway compartment [3]. In randomised controlled trials (RCTs), blood eosinophils and *F*_ENO_ are independently and additively associated with the risk of asthma attacks [4–8]. Importantly, this excess risk is reduced with appropriate treatment at all stages of the disease, be it inhaled corticosteroids (ICS) in mild asthma [9], a higher dose of ICS in moderate asthma [5] or biological agents targeting type-2 cytokines in moderate-to-severe asthma [10, 11]. These observations are consistent with the treatable traits paradigm [12], which emphasises the identification of patient characteristics to guide treatment.

To determine the feasibility of biomarker-stratified risk assessment, we previously derived a prototype Oxford Asthma Attack Risk Scale (ORACLE) (web app available at www.oraclescore.com) [2] using trial-level data from biomarker-stratified RCTs [4–8]. Here, we explore the hypothesis that the excess risk quantified by raised biomarkers in the prototype ORACLE is equivalent to the benefits of anti-inflammatory treatment observed in the derivation trials.

We performed a trial-level analysis of four RCTs in mild [9], moderate [5] and moderate-to-severe asthma [10, 11] comparing biomarker-stratified observed *versus* ORACLE-predicted effects of anti-inflammatory treatments (ICS or type-2 targeting biologics) on annualised severe asthma attack rates. Severe asthma attacks were defined as acute asthma requiring ≥3 days of systemic corticosteroids and/or hospitalisation [2].

Observed annualised severe asthma attack rates of patients randomised to control and active arms were extracted from biomarker-stratified analyses of the Novel START (as-needed salbutamol *versus* low-dose regular or as-needed ICS) [4], CAPTAIN (fluticasone furoate 100 *versus* 200 μg·day^−1^-containing arms) [5], QUEST (placebo *versus* dupilumab) [6, 8] and DREAM (placebo *versus* mepolizumab) [6] studies.

Several assumptions were made during the data collection that are consistent with those used to derive the prototype ORACLE [2]. For both the Novel START [4] and the CAPTAIN trials [7], data of patients with a baseline *F*_ENO_ of 20– <50-ppb were regrouped into the 25– <50-ppb categories, as the difference of 5 ppb in *F*_ENO_ is not clinically relevant [13]. For Novel START [4], only the percentage of patients with one or more severe attack(s) in the 52 weeks of follow-up was reported so an annualised rate was imputed as −log_10_(1−%incidence).

A raw predicted rate was calculated by applying the prototype risk scale parameters in proportion to the reported clinical characteristics of each trial's control-arm population [2]. “Control” and “active” arms’ predicted biomarker-stratified attack rates were calculated based on our hypothesis that the type-2 anti-inflammatory treatment effect in type-2 high asthma (baseline blood eosinophils ≥0.15×10^9^ per L and/or *F*_ENO_ ≥25 ppb) is equivalent to the difference in predicted annualised asthma attack rate in any-biomarker-high stratum *versus* biomarker-low stratum (blood eosinophils <0.15×10^9^ per L and *F*_ENO_ <25 ppb). We further assumed that there would be no anti-inflammatory treatment effect in patients with low baseline biomarkers.

For each trial, observed and predicted rate ratios were calculated between control and active arm attack rates in patients with any raised type-2 biomarker at baseline (blood eosinophils ≥0.15×10^9^ per L and/or *F*_ENO_ ≥25 ppb) and those with none (blood eosinophils <0.15×10^9^ per L and *F*_ENO_ <25 ppb).

The control *versus* treatment arm rate ratios calculated for the observed and predicted biomarker-stratified data were tabulated across individual trials. The main outcome was the comparison of the frequency-weighted mean rate ratio for all observed *versus* predicted treatment effects.

The observed *versus* ORACLE-predicted biomarker-stratified annual asthma attack rates and anti-inflammatory treatment benefits are shown in table 1. For the 3925 patients with any type-2 biomarker high at baseline, the observed *versus* predicted frequency-weighted mean rate ratios were 0.59 *versus* 0.58; the corresponding percentage reductions in asthma attacks were 41% and 42%, respectively. In contrast, the 814 patients with both biomarkers low at baseline had observed *versus* predicted rate ratios of 0.86 *versus* 1.00; the corresponding percentages reduction in asthma attacks were 14% and 0%, respectively. Finally, an exploratory analysis of the 243 patients with both biomarkers very high (*i.e.* blood eosinophils ≥0.30×10^9^ per L and *F*_ENO_ ≥50 ppb), restricted to the Novel START and CAPTAIN studies due to data availability, confirmed a biomarker-dependent treatment response quantified by the prototype ORACLE (observed *versus* predicted percentages reduction in asthma attacks: 69% *versus* 72%).

**TABLE 1:** Predicted *versus* observed impact of anti-inflammatory treatments according to baseline biomarkers

	**Included trial, control *versus* active intervention**				**Weighted mean reduction control *versus* active**
	**Novel START, salbutamol *versus* any low-dose ICS** ^#**,**¶^	**CAPTAIN, FF100 *versus* FF200** ^#^	**QUEST, placebo *versus* dupi200**	**DREAM, placebo *versus* any mepo**	
**Type-2 low: blood Eos <0.15×10^9^ per L and *F*_ENO_ <25 ppb**	n=18 *versus* 60	n=194 *versus* 211	n=106 *versus* 139	n=23 *versus* 63	n=341 *versus* 473
Observed					
Control arm	0.05	0.27	0.56	1.98	
Active arm	0.03	0.22	0.58	1.71	
Reduction	41%	20%	−4%	14%	14%
Predicted					
Control arm	0.07	0.19	0.53	0.74	
Active arm	0.07	0.19	0.53	0.74	
Reduction	0%	0%	0%	0%	0%
**Type-2 high: blood Eos ≥0.15×10^9^ per L or *F*_ENO_ ≥25 ppb**	n=201 *versus* 377	n=903 *versus* 909	n=514 *versus* 484	n=145 *versus* 392	n=1763 *versus* 2162
Observed					
Control arm	0.05	0.40	1.07	2.46	
Active arm	0.03	0.26	0.41	1.19	
Reduction	35%	35%	62%	52%	41%
Predicted					
Control arm	0.13	0.32	0.93	1.28	
Active arm	0.07	0.19	0.53	0.74	
Reduction	42%	42%	42%	42%	42%
**Type-2 very high: blood Eos ≥0.30×10^9^ per L and *F*_ENO_ ≥50 ppb**	n=51 *versus* 54	n=67 *versus* 71	NA	NA	n=118 *versus* 125
Observed					
Control arm	0.13	0.62	NA	NA	
Active arm	0.02	0.25	NA	NA	
Reduction	81%	60%	NA	NA	69%
Predicted					
Control arm	0.26	0.65	1.88	2.60	
Active arm	0.07	0.19	0.82	1.14	
Reduction	72%	72%	72%	72%	72%

Data are presented as annual severe asthma attack rate unless otherwise stated. Data from [4–6, 8], applied to the prototype scale reported in [2]. ICS: inhaled corticosteroids; FF*x*: fluticasone furoate *x* μg·day^−1^; dupi200: dupilumab 200 mg over 2 weeks; mepo: mepolizumab; Eos: eosinophils; *F*_ENO_: exhaled nitric oxide fraction; NA: not available. ^#^: data of patients with a baseline *F*_ENO_ <20 ppb were regrouped into the <25-ppb group, as the difference of 5 ppb in *F*_ENO_ is not clinically relevant [13]; ^¶^: only the percentage of patients with one or more severe attack(s) in the 52 weeks of follow-up was reported so a rate was imputed as −log_10_(1−%incidence).

We found, using trial-level data, that the prototype ORACLE scale may quantify the excess risk conferred by raised biomarkers which is removed by type-2 anti-inflammatory therapy in trial populations. As is the case with cardiovascular risk and management, the relative treatment benefit associated with these biomarkers was consistent across populations but the absolute treatment benefit conferred by type-2 airway inflammation was greater in a population with higher baseline biomarkers and background risk. This information may help doctors and patients make predictions about the likely benefit of type-2 anti-inflammatory treatment to prevent asthma attacks.

To our knowledge, this analysis is the first to suggest a potential theragnostic (*i.e.* predicting treatment responsiveness) utility of a risk prediction model in asthma. Similarities in the visual display, predictive value and utility of cardiovascular risk charts and the prototype ORACLE [2] can be drawn; these are not accidental. Just as high blood pressure and cholesterol levels are regularly assessed to estimate and to prevent the risk of heart attacks, we propose that blood eosinophils and *F*_ENO_ are airway equivalents that measure the modifiable risk of asthma attacks. The demonstration that the ORACLE framework has prognostic and theragnostic value supports our efforts to derive and validate a more robust ORACLE using individual-participant control arm data [14].

We emphasise that our estimations of treatment benefits were derived from trial-level analyses involving several assumptions and that, although promising, several deficiencies mean the prototype ORACLE is not yet validated for clinical practice. First, we were unable to calculate confidence intervals due to regrouping of trial arms and biomarker strata. We thus assessed the theragnostic value of ORACLE based on point estimates, clinical significance [15] and the positive results of anti-inflammatory treatments in type-2 high trial populations [4–6]. Second, our analyses were performed using data from four of the eight RCTs included in the prototype derivation [2] because the other derivation RCTs did not report on the composite biomarker definitions of interest. Despite a systematic review of the literature [14], it was not possible to find external trials reporting the appropriate composite biomarker-stratified subgroups’ control and active treatment attack rates in a manner that allows ORACLE-predicted rates to be calculated. Third, we concede that the estimation of the theragnostic utility of ORACLE in mild asthma trial populations was less precise because of small patient numbers and the rarity of the outcome of interest. Although there was a discrepancy between observed and predicted treatment benefits in mild asthma with low type-2 biomarkers, we still consider that the prognostic importance of blood eosinophils remains relevant in this patient group [4]. Furthermore, the concordance between observed *versus* predicted treatment benefits across moderate-to-severe type-2 low and type-2 high asthma supports the notion that blood eosinophils and *F*_ENO_ are especially useful to gauge the potential benefits of anti-inflammatory treatment beyond low-dose ICS. Fourthly, we assessed different anti-inflammatory treatments in asthma of different severities with and without long-acting bronchodilators, which reduces internal validity for specific combinations, although enhances external validity for anti-inflammatory dosing. Finally, the prototype ORACLE's predictions are based on biomarkers measured at stable state; their value at the time of an exacerbation remains unclear.

To conclude, the prototype ORACLE shows potential to quantify the excess risk of asthma attacks in type-2 high asthma, which is removed by anti-inflammatory therapy. Such a scale discriminating between high-risk/high-stake and low-risk/low-stake asthma is needed in clinical practice, where anti-inflammatory treatment can have a very positive impact when targeted appropriately but can also be escalated without any predictable benefit.
